# Photoaccelerated energy transfer catalysis of the Suzuki–Miyaura coupling through ligand regulation on Ir(iii)–Pd(ii) bimetallic complexes†

**DOI:** 10.1039/d0ra08547b

**Published:** 2020-11-25

**Authors:** Su-Yang Yao, Man-Li Cao, Xiu-Lian Zhang

**Affiliations:** Department of Chemistry, Guangdong University of Education Guangzhou 510303 China jsapaper@163.com

## Abstract

Three bimetallic Ir(iii)–Pd(ii) complexes [Ir(ppy)_2_(bpm)PdCl_2_](PF_6_) (ppy = 2-phenylpyridine, 1), [Ir(dfppy)_2_(bpm)PdCl_2_](PF_6_) (dfppy = (4,6-difluorophenyl)pyridine, 2), and [Ir(pq)_2_(bpm)PdCl_2_](PF_6_) (pq = 2-phenylquinoline, 3) were synthesized by using 2,2′-bipyrimidine (bpm) as a bridging ligand. The influences of the cyclometalated ligand at the Ir(iii) center on the photophysical and electrochemical properties as well as photocatalytic activity for the Suzuki–Miyaura coupling reaction under mild conditions were evaluated. The results revealed that complex 3 enables dramatically accelerating the Suzuki–Miyaura coupling reaction under visible light irradiation at room temperature, due to the effective absorption of visible light and appropriate locus of the excited chromophore. Mechanism studies showed that the chromophore [Ir(pq)_2_(bpm)] fragment absorbs visible light to produce the triplet excited state centering on the bridging ligand which boosts the formation of electron rich Pd(ii) units and facilitates the oxidative addition step of the catalytic cycle. Simultaneously, the excited chromophore undergoes energy transfer efficiently to the Pd(ii) reaction site to form the excited Pd(ii) species, resulting in enhancement of Pd(ii) reduction steps of the Suzuki–Miyaura coupling reaction and increasing the reactivity of the catalyst. This provides a new strategy for designing photocatalysts for coupling reaction through altering the cyclometalated ligand to modulate the photophysical properties and the cooperation between two metal units.

## Abstract 2

A series bimetallic catalysts were synthesized. Relationship between the structure of catalysts and catalytic reactivities were studied and improvement of the catalytic efficiency for Suzuki–Miyaura coupling was accomplished by regulating their chromophores.

## Graphical abstract

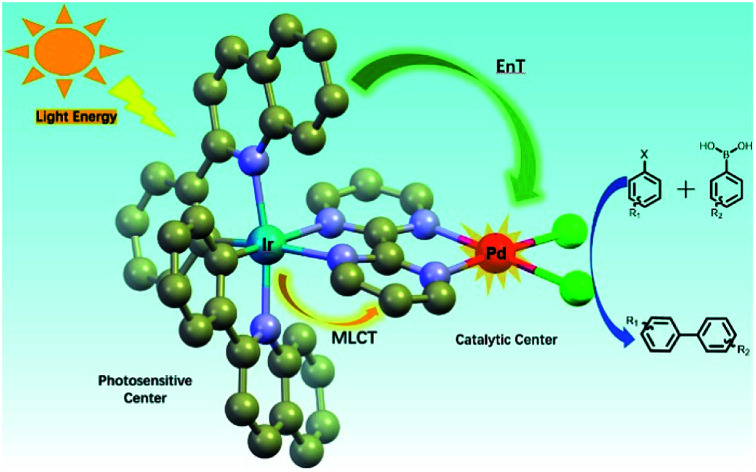

## Introduction

Palladium-catalyzed Suzuki–Miyaura cross-coupling reaction is one of the most powerful protocols for the synthesis of biaryl motifs, which are present in the structures of biologically active compounds and in advanced materials.^1^ Three steps were proposed in the catalytic cycle: oxidative addition, transmetalation reaction and reductive elimination. Among them, the oxidative addition and/or reductive elimination processes have been regarded as the rate limiting steps in the cycle. Therefore, significantly efforts have been devoted during the past decade to accelerate the reaction *via* the introduction of electron-rich and bulky ligands, such as phosphine,^2^ N-heterocyclic carbenes,^3^ palladacycles,^4^ and others.^5^ During these processes, the ligand with strong σ-donor character facilitates the oxidative addition and the bulky ligand enhances the reductive elimination of the product. Moreover, the electron-rich ligand stabilizes the activated Pd center and prevents the Pd black formation in the reaction cycle. However, most of these protocols suffer from high catalyst loading, hazardous solvents, and harsh conditions. Thus, the development of efficient catalytic system is still an ongoing pursuit.

Visible-light catalytic reactions have shown great potential in organic synthesis under mild conditions because of its benign environmental impact and sustainability.^6^ Visible light energy harvested by photocatalysis enable to cooperate with transition metal efficiently such as the generation of excited-state traditional transition metal catalysts which exhibit incredible catalytic potential and remain unexplored.^7^ Therefore, merging chromophore with transition metal catalysts has become a powerful strategy for expanding the synthetic application of visible-light catalysis and has led to the discovery of new reactions, which are not easily accessible in the single catalytic system.^8^ Recently, nanostructured Au–Pd catalysts have shown high efficient activity for the Suzuki–Miyaura coupling reaction under light irradiation due to the electronic heterogeneity at the Au–Pd surface and the energy absorbed from the incident light.^9^ Apart from plasmonic metals, mesoporous g-C_3_N_4_,^10^ conjugated microporous polymers^11^ and inorganic semiconductor,^12^ well-established visible light nonmetal photocatalyst, were also used as photoactive anchoring support for Pd nanoparticles as a model of a Mott–Schottky accelerated the Suzuki reaction. Moreover, cyclometalated Ir(iii) and polypyridyl Ru(ii) complexes have been demonstrated as ideal visible light photosensitizers due to an intense visible light absorption, long triplet lifetimes and outstanding photostability as well as tuneable photophysical properties,^13^ and were widely applied in organic synthesis.^6,8^ However, the application of these kinds of complexes as photocatalysts to accelerate the Suzuki reaction is still scarce.^14,15^ The photoacceleration reaction mechanism, for example through electron or energy transfer (ET or EnT), is still unclear.^16^

**Scheme 1:**
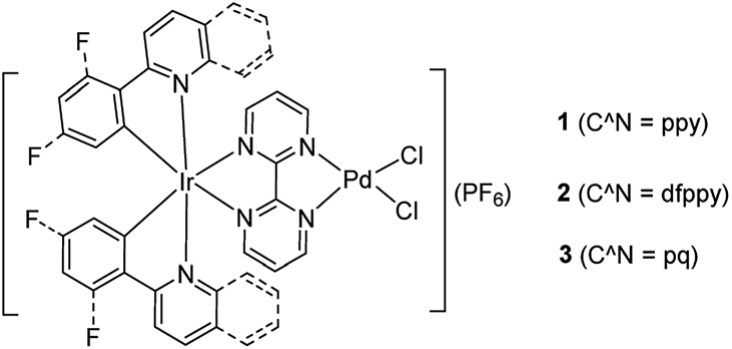
Chemical structures of the bimetallic photocatalysts.

Inspired by the works of Akita's group in promoted polymerization of styrene and vinyl ethers catalyzed by Ru/Ir–Pd photocatalysts^17^ and Yamashita's about enhanced the Suzuki reaction catalyzed by an Ru–Pd bimetallic photocatalyst under visible-light irradiation,^15^ we pay our attention to the Ir–Pd bimetallic photocatalysts. Compared with the Ru(ii) polypyridyl complexes, the cyclometalated Ir(iii) complexes feature high intersystem-crossing (ISC) efficiency, high excited energy and outstanding photostability.^13^ The photocatalysts consisting of a cyclometalated Ir(iii) unit as a chromophore and a Pd(ii) unit as a reaction site are connected *via* a bridging ligand, as shown in Scheme 1. Upon irradiation with visible light, the cyclometalated Ir(iii) complexes would absorb light to produce the excited state which would cooperate with Pd(ii) reaction site through ET or EnT process efficiently and thereby impact on its reaction activity. Here, the synthesis and characterization of Ir–Pd bimetallic complexes [Ir(ppy)_2_(bpm)PdCl_2_](PF_6_) (ppy is 2-phenylpyridine and bpm is 2,2′-bipyrimidine, 1), [Ir(dfppy)_2_(bpm)PdCl_2_](PF_6_) (dfppy is (4,6-difluorophenyl)pyridine, 2), and [Ir(pq)_2_(bpm)PdCl_2_](PF_6_) (pq is 2-phenylquinoline, 3) are reported. Moreover, the photocatalytic activities of these complexes for the Suzuki–Miyaura coupling reaction are also evaluated under visible light irradiation at room temperature. By means of altering of the cyclometalated ligand, we find that catalyst 3 exhibits highly photocatalytic activity for the Suzuki–Miyaura coupling reaction under visible light irradiation *via* EnT from the Ir(iii) chromophore to the Pd(ii) reaction site.

## Experimental

### Materials and general methods

All chemicals were commercially available and used as purchased unless otherwise noted. All manipulations were carried out under an Ar atmosphere unless otherwise noted. Complexes [Ru(bpy)_2_(bpm)](PF_6_)_2_ (bpy is 2,2′-bipyridne), [Ru(bpy)_2_(bpm)PdCl_2_](PF_6_)_2_, [Ir(ppy)_2_(MeCN)_2_](PF_6_), [Ir(dfppy)_2_(MeCN)_2_](PF_6_), [Ir(pq)_2_(MeCN)_2_](PF_6_), and [Ir(pq)_2_(bpy)](PF_6_) were synthesized according to the literatures.^18–21^ Column chromatography was performed with silica gel (300–400 mesh) under low light. Elemental (C, H, and N) analyses were carried out on an Elementar Vario EL analyzer. Electrospray ionization mass spectra (ESI-MS) were obtained on a Thermo LCQ DECA XP mass spectrometer. ^1^H and ^13^C NMR spectra were recorded with a Bruker AV 300 or AV 400 spectrometer using the solvent as an internal standard. Electronic absorption spectra were obtained on a PERSEE TU-1901 UV-vis spectrophotometer. Emission spectra and lifetimes (*τ*) were performed on an FLS920 fluorescence lifetime and steady-state spectrometer at room temperature. Quantum yields (*Φ*_r_) in N_2_ equilibrated DCM solution were determined on an FLS920 fluorescence spectrophotometer using Ru(bpy)_3_(PF_6_)_2_ in N_2_ equilibrated dichloromethane (DCM) solution (*Φ*_r_ = 0.059) as a standard.^22^ Cyclic voltammetric (CV) experiments were performed with a CH Instruments 730C potentiostat. All the measurements were carried out at room temperature in acetonitrile solutions with a sample concentration approximately 1 mM and using 0.1 M tetrabutylammonium hexafluorophosphate as the supporting electrolyte in 200 mV s^−1^ scan rate. A 3 mm diameter glassy carbon working electrode, a Pt auxiliary electrode, and a Pt-wire reference electrode were used. Oxygen was removed from the solutions by bubbling argon for 20 min. All potentials are reported relative to the ferrocenium/ferrocene couple (Fc^+^/Fc), which was used as an external standard to calibrate the reference electrode. The photo-oxidation experiments were carried out with a blue light-emitting diode (LED) corn lamp (*λ* = 450–470 nm, 45 W) obtained from Shenzhen Taoyuan Technology Co., Ltd as a light source. A 100 W UV lamp (*λ* = 365 nm) was obtained Guangzhou Xingchuang Technology Co., Ltd. A green light-emitting diode (LED) corn lamp (*λ* = 510–528 nm, 80 W) obtained from Shenzhen Nuoguan Technology Co., Ltd.

### General procedure for the synthesis of [Ir(C^N)_2_(bpm)](PF_6_) complexes

[Ir(C^N)_2_(MeCN)_2_](PF_6_) (0.12 mmol) and bpm (38 mg, 0.24 mmol) were dissolved into DCM (15 mL). Then, 2 equiv. of KPF_6_ was added to the solution. The solution was stirred at room temperature for 1 h. The solvent was removed under reduced pressure and the raw product was purified by a silica column chromatography using a DCM/MeOH mixture (100 : 1, v/v) as an eluent.

#### For [Ir(ppy)_2_(bpm)](PF_6_)

Yield, 87%, 83 mg. Anal. calcd for C_30_H_22_IrN_6_PF_6_: C 44.83, H 2.76, N 10.46; found: C 44.65, H 2.82, N 10.81%. ESI-MS: *m*/*z* = 658 [M − PF_6_]^+^. ^1^H NMR (400 MHz, CD_3_CN-*d*_3_): *δ* 9.22 (d, *J* = 2.6 Hz, 2H), 8.23 (d, *J* = 5.4 Hz, 2H), 8.10 (d, *J* = 8.1 Hz, 2H), 7.91 (d, *J* = 7.9 Hz, 2H), 7.84 (d, *J* = 7.7 Hz, 2H), 7.76 (d, *J* = 5.4 Hz, 2H), 7.68 (t, *J* = 5.2 Hz, 2H), 7.09 (t, 4H), 6.96 (t, 2H), 6.29 (d, *J* = 7.5 Hz, 2H). ^13^C NMR (101 MHz, CD_3_CN-*d*_3_): *δ* 167.09, 162.05, 159.58, 157.68, 149.86, 147.91, 144.06, 138.86, 131.52, 130.41, 125.40, 124.96, 123.68, 122.95, 119.99.

#### For [Ir(dfppy)_2_(bpm)](PF_6_)

Yield, 85%, 89 mg. Anal. calcd for C_30_H_18_IrN_6_PF_10_: C 41.15, H 2.07, N 9.60; found: C 41.01, H 2.32, N 9.31%. ESI-MS: *m*/*z* = 730 [M − PF_6_]^+^. ^1^H NMR (400 MHz, CD_3_CN-*d*_3_): *δ* 9.26 (d, *J* = 4.5 Hz, 2H), 8.36 (d, *J* = 8.3 Hz, 2H), 8.29 (d, *J* = 5.3 Hz, 2H), 7.97 (t, 2H), 7.78 (d, *J* = 5.6 Hz, 2H), 7.72 (t, 2H), 7.15 (t, 2H), 6.75 (t, 2H), 5.75 (d, *J* = 6.8 Hz, 2H). ^13^C NMR (101 MHz, CD_3_CN-*d*_3_): *δ* 164.62, 163.33, 162.55, 162.08, 161.69, 160.10, 159.96, 158.14, 151.64, 150.26, 139.85, 125.55, 124.12, 123.95, 123.75, 113.78, 99.15.

#### For [Ir(pq)_2_(bpm)](PF_6_)

Yield, 88%, 95 mg. Anal. calcd for C_38_H_26_IrN_6_PF_6_: C 50.50, H 2.90, N 9.30; found: C 50.21, H 2.77, N 9.37%. ESI-MS: *m*/*z* = 759 [M − PF_6_]^+^. ^1^H NMR (400 MHz, CD_2_Cl_2_-*d*_2_): *δ* 9.06 (d, *J* = 2.6 Hz, 2H), 8.55 (d, *J* = 3.4 Hz, 2H), 8.40–8.26 (m, 4H), 8.14 (d, *J* = 7.7 Hz, 2H), 7.82 (d, *J* = 7.9 Hz, 2H), 7.68 (t, 2H), 7.44 (t, 2H), 7.29 (t, 2H), 7.18 (d, *J* = 8.9 Hz, 2H), 7.11 (t, 2H), 6.92 (t, 2H), 6.66 (d, *J* = 7.7 Hz, 2H). ^13^C NMR (101 MHz, CD_2_Cl_2_-*d*_2_): *δ* 169.56, 161.28, 159.67, 154.59, 147.97, 147.09, 145.26, 140.45, 134.82, 131.39, 131.13, 129.49, 127.93, 127.40, 127.05, 124.73, 124.54, 123.74, 117.81.

### General procedure for the synthesis of Ir–Pd bimetallic complexes

[Ir(C^N)_2_(bpm)](PF_6_) (0.039 mmol) and Pd(MeCN)_2_Cl_2_ (10 mg, 0.039 mmol) were added to 2 mL DCM. Then the mixture was stirred at room temperature for an appropriate time (monitored by ^1^H NMR). The solvent was removed under reduced pressure, affording quantitative product without further purification.

#### For 1

The reaction time is 3.5 h. Yield, 99%, 36 mg. Anal. calcd for C_30_H_22_Cl_2_IrN_6_PF_6_Pd: C 36.73, H 2.26, N 8.57. Found: C 36.55, H 2.34, N 8.69%. ESI-MS: *m*/*z* = 837 [M − PF_6_]^+^. ^1^H NMR (400 MHz, CD_3_CN-*d*_3_): *δ* 9.46 (d, *J* = 7.5 Hz, 2H), 8.38 (d, *J* = 7.2 Hz, 2H), 8.10 (d, *J* = 8.1 Hz, 2H), 7.98–7.92 (m, 4H), 7.89 (d, *J* = 5.8 Hz, 2H), 7.84 (d, *J* = 5.8 Hz, 2H), 7.16 (t, 2H), 7.11 (t, 2H), 6.97 (t, 2H), 6.23 (d, *J* = 8.2 Hz, 2H). ^13^C NMR (75 MHz, CD_3_CN-*d*_3_): *δ* 166.40, 165.14, 159.42, 157.24, 151.76, 144.03, 143.64, 139.23, 131.34, 130.47, 128.36, 124.96, 123.97, 123.67, 119.99.

#### For 2

The reaction time is 6 h. Yield, 99%, 41 mg. Anal. calcd for C_30_H_18_Cl_2_IrN_6_PF_10_Pd: C 34.22, H 1.72, N 7.98. Found: C 34.51, H 1.54, N 7.75%. ESI-MS: *m*/*z* = 907 [M − PF_6_]^+^. ^1^H NMR (400 MHz, CD_3_CN-*d*_3_): *δ* 9.45 (d, *J* = 4.1 Hz, 2H), 8.46 (d, 2H), 8.35 (d, *J* = 5.7 Hz, 2H), 8.01 (t, 2H), 7.97 (t, 2H), 7.93 (d, *J* = 5.7 Hz, 2H), 7.21 (t, 2H), 6.79 (ddd, *J* = 12.0, 9.4, 2.3 Hz, 2H), 5.69 (dd, *J* = 8.7, 2.4 Hz, 2H). ^13^C NMR (101 MHz, CD_3_CN-*d*_3_): *δ* 164.80, 164.34, 162.76, 162.37, 161.91, 159.98, 159.66, 157.79, 152.12, 146.85, 140.18, 128.40, 124.41, 123.94, 123.74, 113.94, 99.87.

#### For 3

The reaction time is 2 h. Yield, 99%, 42 mg. Anal. calcd for C_38_H_26_Cl_2_IrN_6_PF_6_Pd: C 42.22, H 2.42, N 7.77. Found: C 42.31, H 2.71, N 7.85%. ESI-MS: *m*/*z* = 935 [M − PF_6_]^+^. ^1^H NMR (400 MHz, CD_2_Cl_2_-*d*_2_): *δ* 9.08 (t, 2H), 8.65 (d, *J* = 5.6 Hz, 2H), 8.34 (d, *J* = 8.8 Hz, 2H), 8.26 (d, *J* = 8.8 Hz, 2H), 8.11 (d, *J* = 7.7 Hz, 2H), 8.02 (t, 2H), 7.81 (d, *J* = 7.7 Hz, 2H), 7.45 (t, 2H), 7.29 (t, 2H), 7.21 (t, 2H), 6.96 (d, *J* = 8.9 Hz, 2H), 6.91 (t, 2H), 6.61 (d, *J* = 7.6 Hz, 2H). ^13^C NMR (101 MHz, CD_2_Cl_2_-*d*_2_): *δ* 168.71, 163.29, 157.88, 156.01, 147.03, 145.04, 144.09, 140.84, 135.10, 132.40, 131.08, 129.57, 128.10, 127.59, 127.38, 127.27, 124.59, 124.51, 117.69.

### General procedure for photocatalysis of the Suzuki–Miyaura coupling reaction

Pd(MeCN)_2_Cl_2_ (2.5 mol%) and corresponding [Ir(C^N)_2_(bpm)](PF_6_) complex (2.5 mol%) were pre-reacted in 2 mL DCE for corresponding hours in a Schlenk tube, then PPh_3_ (5 mol%), benzene halide (0.15 mmol), arylboronic acid (0.3 mmol), and Cs_2_CO_3_ (100 mg, 0.3 mmol) were added to the tube. The tube was degassed three times, then a solution of 1,2-dichloroethane–ethanol (DCE–EtOH, 2 mL, 1 : 1, v/v) was injected into the tube. The reaction was conducted at room temperature under Ar atmosphere with irradiation of a 45 W blue LED from *ca.* 10 cm for an appropriate time (monitored by TLC). The reaction products were purified by silica gel chromatography using hexane/ethyl acetate (7/1, v/v) as an eluent, which were characterized by ^1^H NMR (see the ESI†).

## Results and discussion

### Synthesis of Ir(iii)–Pd(ii) bimetallic complexes

**Scheme 2:**
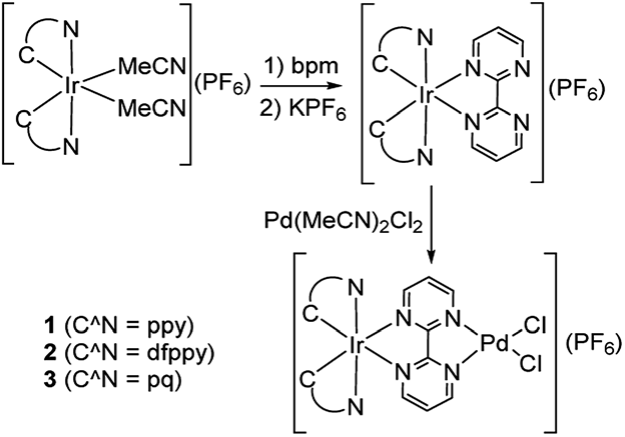
Synthesis of Ir(iii)–Pd(ii) bimetallic complexes.

To observe the impact of cyclometalated ligand on the photophysical properties and photocatalytic activity of the Ir(iii)–Pd(ii) bimetallic complexes, three bimetallic complexes with different cyclomelated ligands (ppy, dfppy, and pq) were designed and synthesized, as shown in Scheme 2. The mononuclear bpm complexes were synthesized in good yields of 85–88% by reaction of the precursors [Ir(C^N)_2_(MeCN)_2_](PF_6_) with bpm ligand at room temperature. The complexes were characterized by ^1^H NMR, ^13^C NMR spectroscopy and mass spectrometry as well as elemental analysis. Furthermore, the bimetallic complexes were obtained by the reaction of bpm complexes with Pd(MeCN)_2_Cl_2_ at room temperature in almost quantitative yields. The bimetallic complexes were also identified on the basis of NMR spectroscopy and mass spectrometry.

### Photophysical properties

**Fig. 1:**
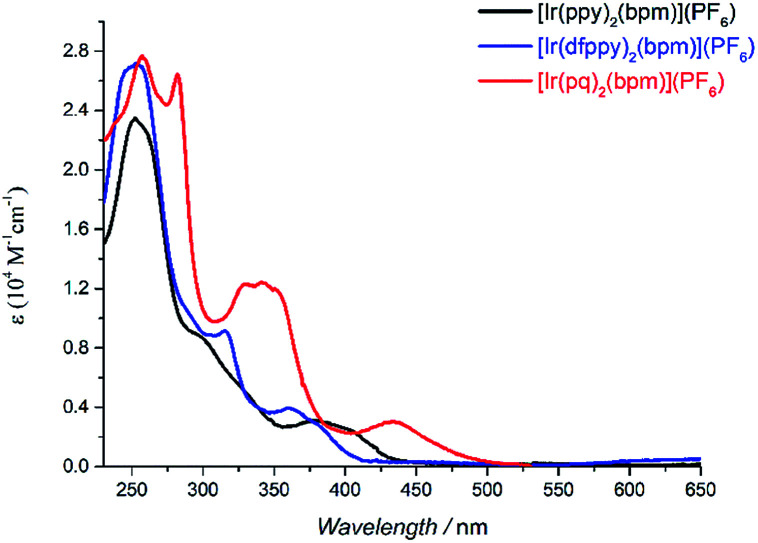
UV-vis spectra of mononuclear Ir(iii) complexes in DCM solution (5 × 10^−5^ M).

Table 1 summarizes the main photophysical properties of the mononuclear and bimetallic complexes in DCM solution at room temperature. As depicted in Fig. 1, the absorption spectra for the mononuclear complexes are comprised of three main absorption bands. An intense band is observed at 250–281 nm, which can be attributed to the spin-allowed π–π* ligand centered (LC) transitions. The moderately intense band at 300–350 nm is mainly attributed to the spin-allowed metal-to-ligand charge transfer (^1^MLCT).^23^ While the weaker absorption band at 415 nm for [Ir(ppy)_2_(bpm)](PF_6_), 390 nm for [Ir(dfppy)_2_(bpm)](PF_6_), and 433 nm for [Ir(pq)_2_(bpm)](PF_6_) mainly come from the spin-forbidden ^3^MLCT transitions according to related reports on [Ir(pq)_2_(bpy-CH_2_NH_2_)](PF_6_) and [Ir(pq)_2_(bpy)][B(5FPh)_4_] (where bpy-CH_2_NH_2_ is 4-aminomethyl-4′-methyl-2,2′-bipyridine and B(5FPh)_4_ is tetrakis(2,3,4,5,6-pentafluorophenyl)borate).^23^ Compared to [Ir(ppy)_2_(bpm)](PF_6_), the introduction of F groups in phenyl and substitution pyridine with quinoline leads to a blue and red shift, respectively. The former can be attributed to the remarkable metal-based MOs stabilization caused by the introduction electron-withdrawing groups on phenyl of the cyclometalated ligand. And the main reason for the latter is the presence of the quinoline moiety which can stabilize the unoccupied MOs. These are also supported by DFT calculations of [Ir(dfppy)_2_(bpm)]^+^ and [Ir(pq)_2_(bpm)]^+^ as shown in Scheme S1.† Moreover, the broad MLCT bands for [Ir(ppy)_2_(bpm)](PF_6_) and [Ir(pq)_2_(bpm)](PF_6_) complexes suggest that they are capable of harvesting light energy from UV to visible region.

**Table 1:** Photophysical data for mononuclear Ir(iii) and their corresponding Ir(iii)–Pd(ii) complex

Complex	*λ*_abs_ a [nm] (*ε* [10^4^ M^−1^ cm^−1^])	*λ*_em_ b [nm]	*τ* b [ns]	*Φ*_PL_ b [×10^−3^]	*E*_ox_ c [V]	*E*_red_ c [V]
[Ir(ppy)_2_(bpm)]^+^	253(2.3), 300(0.86), 379(0.31), 415(sh)	700	7	6.7	1.30	−1.56, −1.93
[Ir(dfppy)_2_(bpm)]^+^	251(2.7), 315(1.1), 362(0.4), 390(sh)	611	288	107	1.75	−1.55, −2.20
[Ir(pq)_2_(bpm)]^+^	252(2.6), 281(2.6), 350(1.2), 433(0.3)	687	12	10.3	1.38	−1.50, −1.93
1	270(2.2), 312(sh), 375(0.42)	556	n.d.	0.37	1.38	−2.0, −2.38
2	255(sh), 313(1.1), 359(sh)	683	n.d.	8.1	1.65	−2.50
3	279(3.4), 330(1.3), 356(1.3), 363(1.3), 427(0.4)	584	n.d.	0.44	1.35	−1.99, −2.24

a In deaerated DCM solution (5 × 10^−5^ M).

b In deaerated DCM solution (2 × 10^−4^ M, *λ*_ex_ = 405 nm).

c Cyclic voltammograms carried out at a scan rate of 200 mV s^−1^, *versus* Fc/Fc^+^ using 0.1 M TBAPF_6_ as a supporting electrolyte in degassed CH_3_CN.

**Fig. 2:**
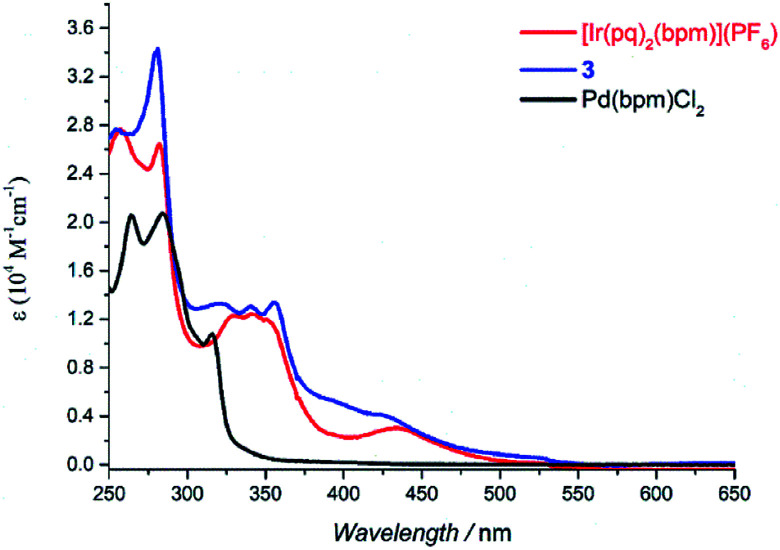
UV-vis spectra of [Ir(pq)_2_(bpm)](PF_6_), 3, and Pd(bpm)Cl_2_ in DCM solution (5 × 10^−5^ M).

**Fig. 3:**
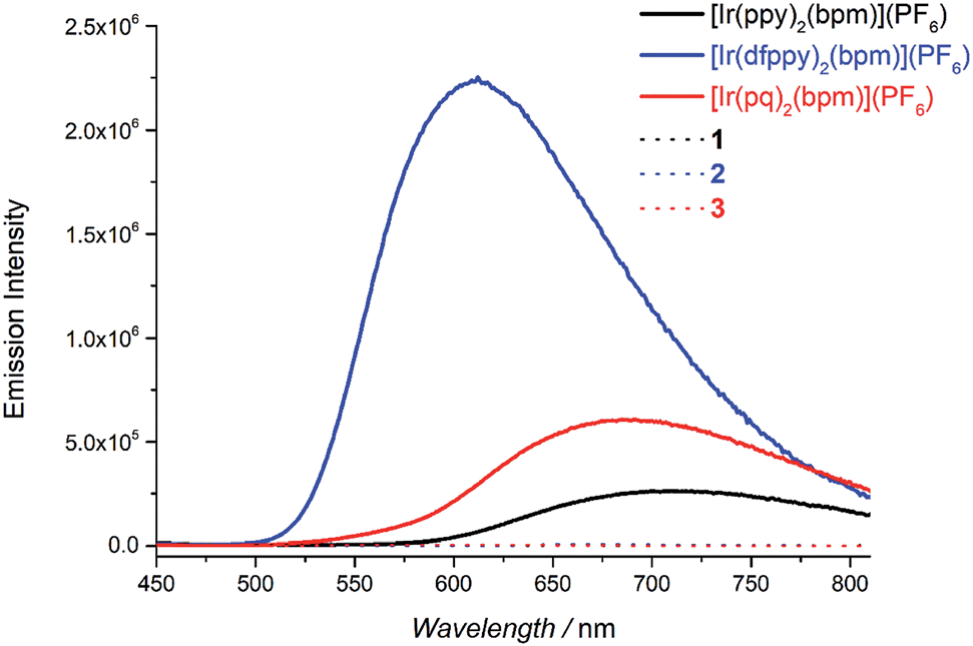
The mononuclear and bimetallic complexes in degassed DCM solution at room temperature (2 × 10^−4^ M, *l*_ex_ = 405 nm).

The absorption spectra of Pd(bpm)Cl_2_, [Ir(pq)_2_(bpm)](PF_6_) and its corresponding Ir(iii)–Pd(ii) complex 3 was shown in Fig. 2. Compared to the mononuclear Ir(iii) complex, binuclear Ir(iii)–Pd(ii) complex 3 present a spectrum which is similar to the combination of the component spectra. However, small changes of the spectrum around 400 nm and 500 nm imply that underlying new electronic transitions occurred between the two components in the bimetallic complex.^24^ The absorption spectra of other Ir(iii)–Pd(ii) bimetallic complexes also exhibit the similar phenomenon as shown in Fig. S1 and S2.† The emission spectra of the complexes were measured in degassed DCM solution at room temperature (*λ*_ex_ = 405 nm), shown in Fig. 3. The mononuclear complexes show an emission with maximum emission band at 700 nm (*Φ*_PL_ = 0.67% and *t* = 7 ns), 611 nm (*Φ*_PL_ = 10.7% and *t* = 288 ns), and 687 nm (*Φ*_PL_ = 1.03% and *t* = 12 ns) for [Ir(ppy)_2_(bpm)](PF_6_), [Ir(dfppy)_2_(bpm)](PF_6_), and [Ir(pq)_2_(bpm)](PF_6_), respectively. The changing of the cyclometalated ligand from ppy to dfppy leads to the emission wavelength significantly blue shift from 700 nm to 611 nm and the triplet state lifetime elongation from 7 ns to 288 ns. These may be attribute to the introduction of F group on cyclometalated ligand which affects the energy of essential molecular orbitals and results in the change of low energy charge transfer states. The emission of [Ir(ppy)_2_(bpm)](PF_6_) and [Ir(pq)_2_(bpm)](PF_6_) can be mainly assigned to ^3^MLCT (dπ(Ir) → π*(bpm)) based transition,^21^ whereas a mixture of ^3^MLCT (dπ(Ir) → π*(dfppy) and dπ(Ir) → π*(bpm)) transitions may show a large contribution to the excited state of [Ir(dfppy)_2_(bpm)](PF_6_).^25,26^ Comparison with the corresponding complexes [Ir(ppy)_2_(bpy)](PF_6_) (*λ*_em_ = 610 nm, *Φ*_PL_ = 0.89%, and *t* = 103 ns),^21^ [Ir(dfppy)_2_(bpy)](PF_6_) (*λ*_em_ = 520 nm, *Φ*_PL_ = 26%, and *t* = 561 ns),^27^ and [Ir(pq)_2_(bpy)](PF_6_) (*λ*_em_ = 560 nm, *Φ*_PL_ = 14%, and *t* = 474 ns),^21^ the emission intensity of the mononuclear complexes are decrease due the presence of two sets of free lone-pairs in the bpm ligand which may cause electronic repulsion, resulting in thermodynamic quenching of the triplet excited-state to some extent. The introduction of Pd(ii) center to the mononuclear complex leads to a drastically luminescent quenching compared with the corresponding mononuclear complex (Fig. 3) which is associated with much faster deactivation channels like electron or energy transfer starting from energy levels above the relaxed ^3^MLCT excited state. To comparison, the emission of a mixture of the mononuclear [Ir(pq)_2_(bpm)](PF_6_) and [Pd(bpm)Cl_2_] complexes was also observed and exhibited a slight quenching (see Fig. S3 in the ESI†), indicating that the intramolecular ET/EnT process from the excited state of Ir(iii) complex to Pd(ii) center is highly efficient.^15,28^ The quantum yields roughly follow in the case of photoluminescence of the binuclear complexes. And the PL intensive quenching of complexes 1 and 3 where the Ir-based ^3^MLCT mainly contributes from the bridged ligand bpm is more effective by a factor of about 20 as compared with complex 2.

### Electrochemistry

Cyclic voltammetry was used to determine the ground state redox potentials of all the Ir(iii) complexes. As listed in Table 1 and Fig. S4,† [Ir(ppy)_2_(bpm)](PF_6_), [Ir(dFppy)_2_(bpm)](PF_6_), and [Ir(pq)_2_(bpm)](PF_6_) showed a quasi-reversible couple at about +1.30 V, +1.75 V, and +1.38 V, respectively, which was assigned to Ir(iv/iii) oxidation.^23*b*^ The more positive oxidation potential for [Ir(dFppy)_2_(bpm)](PF_6_) can be attributed to the introduction of electron-withdrawing F groups on phenyl ring of dfppy ligand, which stabilize the t_2g_ orbital of Ir(iii) ion, resulting in more difficult to lose electron. This is consistent with the observed emission properties. However, the precisely redox data for bimetallic complexes are not obtained because they are electrochemical too unstable to detect (see Fig. S5 in the ESI†).^28^

### Photoacceleration of the Suzuki–Miyaura coupling reaction

The high-efficiently intramolecular ET or EnT from the excited state of the Ir(iii) complex to the Pd(ii) center encouraged us to estimate the photoaccelerated Suzuki–Miyaura coupling reaction using these bimetallic complexes as photocatalysts, where the Ir(iii) fragments act as a chromophore and the Pd(ii) fragments act as a reaction site. We began our study using 4-bromotoluene and phenylboronic acid as model substrates to screen the reaction conditions for the Suzuki–Miyaura coupling reaction in the presence of 3 (2.5 mol%) and PPh_3_ (5 mol%) as catalysts and Cs_2_CO_3_ as a base in an environment-friendly solution EtOH under Ar atmosphere and visible light irradiation at room temperature for 5 h (see Table 2, entry 1). To our delight, the coupling reaction went smoothly under mild conditions, affording the corresponding product in a moderate yield of 63% (entry 1 in Table 2). When MeCN was used as a solvent instead of EtOH, the isolated yield decreased to 46% (entry 2), indicating that a solvent has much impact on the coupling reaction. When DCE was used, the yield increased to 81% (entry 3 in Table 2). These also inspired us to continuously optimize the reaction solvent. It was found that a mixture of DCE–EtOH (3 : 1) is the best one, affording an excellent isolated yield in 93%. The superior function of the mixed solvent may be due to the increased solubility of the reactants and base. Since base has a significant influence on the Suzuki–Miyaura coupling, we then turned our attention to test the impact of base (entries 4–6). The experiments revealed that Cs_2_CO_3_ is the best one. K_2_CO_3_ is slight inferior, affording a moderate yield in 65%, but is superior to Et_3_N. Moreover, the control experiments (entries 6–10) demonstrated that the bimetallic catalyst, base, and PPh_3_ ligand are indispensable to the coupling reaction under mild conditions. The necessary of PPh_3_ ligand may be attributed to the effect of bulk and electron richness at the Pd center, which can accelerate the rates of both oxidative addition and reductive elimination steps. The photocatalytic reaction was significantly suppressed in air, affording only 18% yield. This may be ascribed that the excited state of Ir(iii) complex was easily quenched by O_2_ and failed to cooperate with the Pd(ii) unit to some extent. Meanwhile, the presence of O_2_ also impedes the reductive elimination step of catalytic cycle and both these factors result in a low reactivity. In summary, visible light significantly accelerated the coupling reaction, increasing the yield from 40% to 93%. When the amount of catalyst was decreased to 1.0 mol%, the isolated yield decreased to 81% for 18 h under the identical conditions (see entry 11). To study the relationship between light absorption wavelength and catalytic activity, experiments using 100 W UV lamp (*λ* = 365 nm) and 80 W green LED corn lamp (*λ* = 510–528 nm) as light source were conducted under the same condition (entries 12 and 13). The yield of product under UV lamp was similar to that under blue light. However, the yield of product under green light significantly decreased to 46%. These indicate that the utilizations of blue light and ultraviolet light energy of complex 3 are similar. However, complex 3 cannot fully harness the green light and lead to the low catalytic reactivity which is in accord with its UV-vis spectrum. The action spectrum was shown in Fig. S6.† Furthermore, the photocatalytic activities of the other bimetallic complexes for the Suzuki–Miyaura coupling reaction were also evaluated under the optimal conditions (entries 14–16). When complex 1 and [Ru(bpy)_2_(bpm)PdCl_2_](PF_6_)_2_ were used instead of 3 as catalysts, the yields of the coupling product slightly lowered to 86% and 80%, respectively. These may be related to the stronger absorption of complex 3 in the visible region and the less stability of bimetallic complex 1 compared with complex 3. The yield dramatically decreased to 54% when complex 2 was used as a photocatalyst, which was slightly higher than the catalytic activity of [Pd(bpm)Cl_2_] (47%, see entry 18) indicating that no significantly accelerated coupling reaction was observed in complex 2 under irradiation, even though complex 2 is a more strongly oxidizing photocatalyst. Since the absorption intensity of visible region of complex 2 is weaker than other bimetallic Ir–pd complexes, 100 W UV lamp (*λ* = 365 nm) was used as a light source to irradiate the reaction, however, the yield of the product only slightly increased to 59% (entry 17). Therefore, the different absorption intensity in visible region can be exclude for the main reason to explain the low reactivity of complex 2. Moreover, the different chromophore fragments also result in factors such as structure and redox properties of Ir(iii) unit which may influence the catalytic efficiency without undergoing the photoexcited process. So, it is important to consider these factors as potentially reasons for the low reactivity of complex 2. To verify these options, reaction using catalyst complex 2 under the dark was conducted. However, the yield of product is similar with the reaction when using complex 3 as catalyst under the dark (entries 10 and 19). The similar yield was also observed when using complex 1 as catalyst under the dark (entry 20). These observations imply that the different properties of chromophore fragments cannot lead to the significant different reactivities of catalysts when the Ir(iii) unit is in their ground state and the main reason for the conspicuous difference catalytic reactivity under visible light may attribute to the excited state of the chromophore and the cooperation between Ir(iii)* and Pd(ii) unit. Hence, we speculated that the reason of the dramatically decreased activity of complex 2 may be associated with its major contribution of ^3^MLCT (dπ(Ir) → π*(dfppy)) transition and the EnT process between two metal units (*vide supra*). Compared with complex 2, the ^3^MLCT transition of complex 3 is mainly assigned to dπ(Ir) → π*(bpm) and leads to an excited state which locate on bridging ligand. This kind of locus of excited state on one hand may increase the charge density of the bridge ligand and boost the formation of electron rich Pd unit which could facilitate the oxidative addition step of catalytic cycle.^15,28^ On the other hand, the transition direction from Ir(iii) unit to the bridging ligand which is directly connected with Pd(ii) unit could also facilitate the EnT from the excited state of chromophore to the reaction site through the bridged ligand bpm,^17*a*,28^ resulting in acceleration of the Suzuki–Miyaura coupling reaction. Moreover, we also have a research on the catalytic efficiency between different catalytic system. The yields comes to 70% and 75% respectively when a mixture of [Ir(pq)_2_(bpm)](PF_6_) and Pd(MeCN)_2_Cl_2_(without pre-reacting to form bimetallic complex), and [Ir(pq)_2_(bpy)](PF_6_) and [Pd(bpm)Cl_2_] were used as photocatalysts (entries 21 and 22), indicating that visible light significantly accelerates the Suzuki–Miyaura coupling reaction (compared with the reaction by using [Pd(bpm)Cl_2_] as catalyst in the dark, see entry 18) and the cooperation between intramolecular catalytic system is more efficient compared to the intermolecular system (see entry 4). According to the previous studies,^9–15^ two mechanisms may involve the photoaccelerated Suzuki–Miyaura coupling reaction. A 2-electron transfer from the excited component to the Pd(ii) site generates an active electron rich Pd(0) which facilitates the oxidative addition reaction with aryl halides.^10–12,15^ Alternatively, an EnT from the triplet excited state of chromophore produces highly energetic electrons at the Pd catalytic site, which enhances the coupling reaction.^9*a*,9*b*,14^ To verify our former speculation of the cooperation process between two metal units and gain further insight into the photocatalytic coupling reaction, control experiments were performed to distinguish the mechanism of the photocatalyzed Suzuki–Miyaura coupling reaction. When two equiv. of triethylamine (TEA), which has been demonstrated as an sacrificial electron donor for excited cyclometalated Ir(iii) complexes,^29^ was added to the reaction solution under the optimal conditions (see entry 23), the isolated yield was afforded in 89%, indicating that there is no obvious suppression of the coupling reaction. Then we engaged complex 2 which containing a more strongly oxidizing chromophore as catalyst. To our surprise, the addition of Et_3_N significantly suppressed the reactivity of catalyst (see entry 24) indicating that Et_3_N enable to quench the excited Ir(iii)* center thoroughly and impede the formation of excited Pd(ii)* center through electron transfer from quenched Ir unit to Pd(ii) unit, so the ET process may be ruled out in this case.^30,31,7*b*^ Furthermore, the intense light energy was used to find whether excited Pd(ii)* complex can improve the coupling reaction. The reaction by using [Pd(bpm)Cl_2_] as a photocatalyst under UV lamp irradiation was conducted (see entry 25). To our delight, the isolated yield was indeed increased from 47% to 63% which indicating that the formation of a excited Pd(ii)* state is available through photoexcitation which can indeed enhance the reactivity of the coupling reaction. This mechanistic proof points to an EnT process between two metal units.

**Table 2:** Optimal photocatalytic conditions^a^

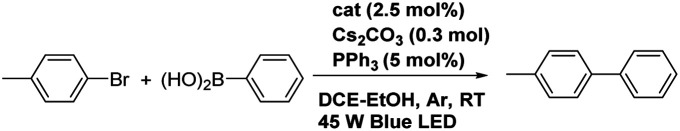			
Entry	Catalyst	Different condition	Yield^b^ (%)
1	3	EtOH	63
2	3	MeCN	46
3	3	DCE	81
4	3	—	93
5	3	K_2_CO_3_	65
6	3	Et_3_N	Trace
7	—	—	0
8	3	No PPh_3_	Trace
9	3	Air	18
10	3	Dark	40
11^c^	3 (1 mol%)	PPh_3_ (2 mol%)	81
12^d^	3	UV lamp	88
13^e^	3	Green LED corn lamp	46
14	1	—	86
15	2	—	54
16^f^	A	—	80
17^d^	2	UV lamp	59
18	[Pd(bpm)Cl_2_]	Dark	47
19	2	Dark	36
20	1	Dark	41
21^g^	B	—	70
22^h^	C	—	75
23^i^	3	2 equiv. Et_3_N	89
24^i^	2	2 equiv. Et_3_N	20
25^d^	[Pd(bpm)Cl_2_]	UV lamp	63

a Conditions: 4-bromotoluene (0.15 mmol), phenylboronic acid (0.3 mmol), catalyst (2.5 mol%), PPh_3_ (5 mol%), Cs_2_CO_3_ (0.3 mmol) in 4 mL of DCE–EtOH (3 : 1) with Ar atmosphere under a 45 W blue LED corn lamp at room temperature for 5 h.

b Isolated yield.

c In 18 h.

d 100 W UV lamp was used as light source.

e 80 W green LED corn lamp was used as light source.

f A = [Ru(bpy)_2_(bpm)PdCl_2_](PF_6_)_2_.

g B = [Ir(pq)_2_(bpm)](PF_6_) + Pd(MeCN)_2_Cl_2_.

h C = [Ir(pq)_2_(bpy)](PF_6_) + Pd(bpm)Cl_2_.

i Additional addition 2 equiv. of Et_3_N.

**Scheme 3:**
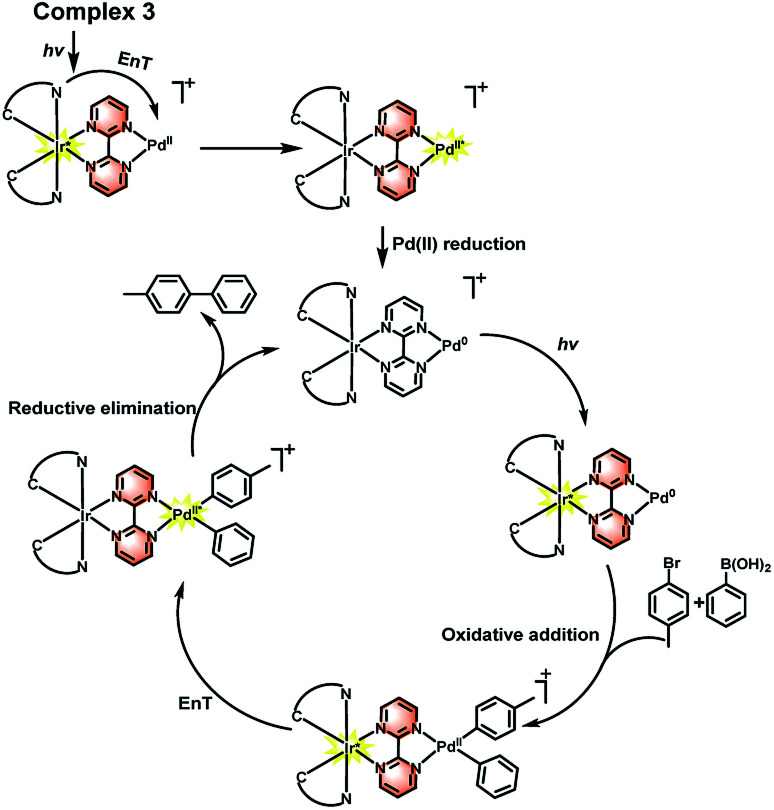
Mechanism of the photoaccelerated C–C coupling reaction.

Therefore, combined with the previous reported mechanism,^32,33^ a photoaccelerated coupling reaction *via* EnT was proposed on the basis of our experiments, as shown in Scheme 3. Under light irradiation, the Ir(iii) complex is converted into a high-energy excited singlet state ^1^Ir(iii)*, which undergoes intersystem crossing to triplet ^3^Ir(iii)* quickly since Ir(iii) complex possesses highly efficient spin–orbit coupling. The ^3^Ir(iii)* can transfer energy to the Pd(ii) center to form a triplet excited state ^3^Pd(ii)*, which enables to enhance Pd(ii) reduction process in the catalytic cycle and increase the formation the C–C crossing coupling product. The similar process has also been demonstrated in the C–C and C–N crossing coupling reactions using Ir(iii) and Ni(ii) bimolecular photocatalysts.^7*b*,7*d*^ Furthermore, the light irradiation may also accelerated the oxidative addition step because the locus of excited state of chromophore which enable to increase the charge density of the bridge ligand and boost the formation of electron rich Pd unit and facilitate the carbon–halogen bond activation.^9*d*,15,34,35^

With the optimal conditions in hand, we further observed the scopes and limitations of this photoaccelerated Suzuki–Miyaura coupling reaction under mild conditions (see Table 3). First, the steric effect on the reaction was investigated. When 2-bromotoluene, 3-bromotoluene, and 3,5-dimethyl-bromobenzene were used as substrates to couple with phenylboronic acid in the standard conditions, the corresponding desired products 4b, 4c, and 4d were afforded in 89%, 93%, and 90% for 8 h, respectively, demonstrating that steric effect of the arylbromide was insensitive to the reaction. When electron-withdrawing 4-acetyl group was introduced on arylbromide, the corresponding product 4e was afforded in an excellent isolated yield of 95% for 4 h, indicating that the electronic effect on arylbromide was sensitive in our case and the electron-deficient substitution was beneficial to the Suzuki–Miyaura coupling reaction. However, when the low activity of 4-acetyl-chlorotoluene was used instead of 4-acetyl-bromotoluene, the coupling reaction became sluggishly, almost no product was observed for 12 h. This case was improved by using PCy_3_ instead of PPh_3_ and elevating the reaction temperature to 60 °C, affording 4e in a moderate yield of 51% for 15 h. Moreover, the scope of phenylboronic acid was also observed. The electron-deficient substitution 4-F and electron-rich substitution 4-methoxy phenylboronic acids efficiently coupled with bromotoluene to provide the corresponding products 4f and 4g in excellent yields of 92% and 93%, respectively, indicating that the electronic effect of phenylboronic acid was insensitive to the coupling reaction. Finally, 1-bromonaphthalene and 2-methyl-1-bromonaphthalene were used as coupling partners with phenylboronic acids, affording the corresponding 4h, 4i, and 4j in yields of 85%, 78%, and 81%, respectively, in 24 h. Moreover, the photoaccelerated coupling reaction for the synthesis of binaphthyl was also observed, because it is a chiral atropisomer along the biaryl axis and important for many biologically active products and potential pharmaceuticals. Indeed, when 2-methyl-1-bromonaphthalene and 2-methoxy-1-bromonaphthalene were used as coupling partners with and 1-naphthylboronic acid in the presence of 3 (4.0 mol%) and PPh_3_ (8.0 mol%) as catalysts, the corresponding products 4k and 4l were afforded in moderate yields of 57% and 55% for 24 h, respectively. The yield significantly enhanced to 80% when PCy_3_ was used instead of PPh_3_ for 5 h, indicating that the visible light accelerated protocol can be used to synthesize binaphthyl compounds.

**Table 3:** Scope of the photoaccelerated Suzuki–Miyaura coupling reaction^a^

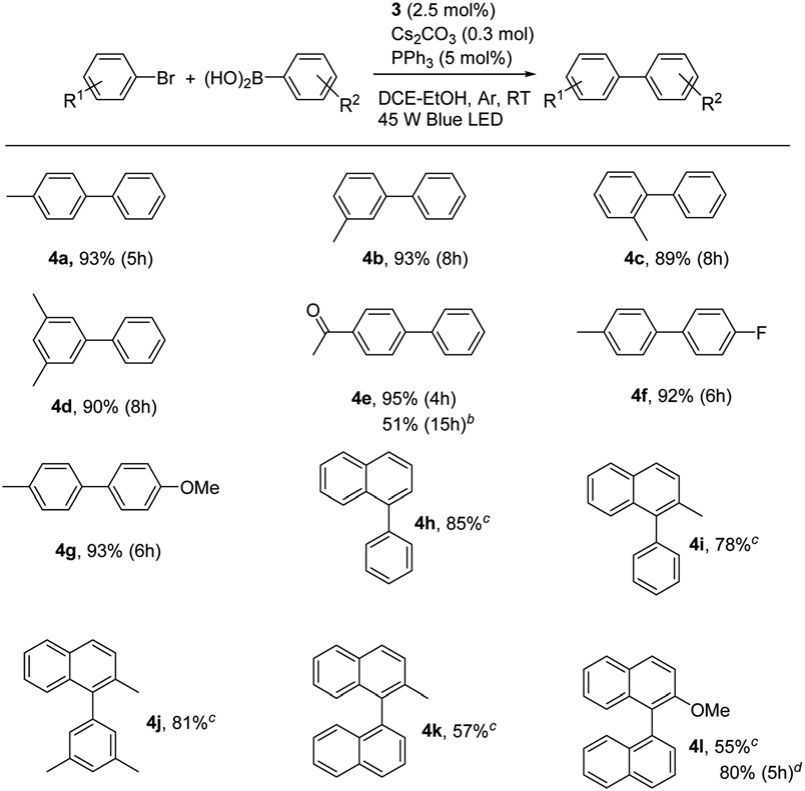

a Conditions: arylbromide (0.15 mmol), arylboronic acid (0.3 mmol), 3 (2.5 mol%), PPh_3_ (5 mol%), Cs_2_CO_3_ (0.3 mmol) in 4 mL of DCE–EtOH (3 : 1) with Ar atmosphere under a 45 W blue LED at room temperature; isolated yield.

b 4-Acetyl-chlorotoluene and PCy_3_ were used instead of 4-acetyl-bromotoluene and PPh_3_ at 60 °C.

c 3 (4.0 mol%) and PPh_3_ (8.0 mol%) were used in 24 h.

d 3 (4.0 mol%) and PCy_3_ (8.0 mol%) were used.

## Conclusions

In summary, a series of bimetallic complexes consisting of a chromophore and a reaction site were synthesized. The influences of the cyclometalated ligand at Ir(iii) center on the photophysical properties and the photocatalytic activity were demonstrated. Through the regulation of cyclometalated ligands at Ir(iii) center, complex 3 could dramatically accelerate the Suzuki–Miyaura coupling reaction under a mild condition. The highly effective absorbing of visible light and appropriate locus of excited chromophore were proved to facilitate the photoaccelerated reaction. Mechanism studies show that the light energy absorbed by Ir(iii) fragment produces the excited chromophore and then undergoes EnT efficiently to the reaction site to form a excited Pd(ii) center *via* the bridging ligand which could accelerate the catalytic activity of complex. This study could provide a further exploration of bimetallic complexes in photocatalyst and a strategy about accelerating the complexes' catalytic efficiency through modifying their structures by affecting the photophysical properties and cooperation between two metal units.

## Conflicts of interest

There are no conflicts to declare.

## Supplementary Material

RA-010-D0RA08547B-s001
