# Supplementary material for: Photoaccelerated energy transfer catalysis of the Suzuki–Miyaura coupling through ligand regulation on Ir(iii)–Pd(ii) bimetallic complexes
Source: RSC Adv. 2020 Nov 25;10(70):42874–82. doi: 10.1039/d0ra08547b (PMC9058252; doi:10.1039/d0ra08547b)
Supplement: RA-010-D0RA08547B-s001 [file RA-010-D0RA08547B-s001.pdf]

Electronic Supplementary Material (ESI) for RSC Advances.
This journal is © The Royal Society of Chemistry 2020

Supporting Information

for

Photoacceleration of the Suzuki-Miyaura coupling through ligand-regulation on Ir(III)-Pd(II) bimetallic complexes

Su-Yang Yao[a], Xiu-Lian Zhang [a], and Man-Li Cao*[a]

[a]Department of Chemistry, Guangdong University of Education, Guangzhou 510303, China.

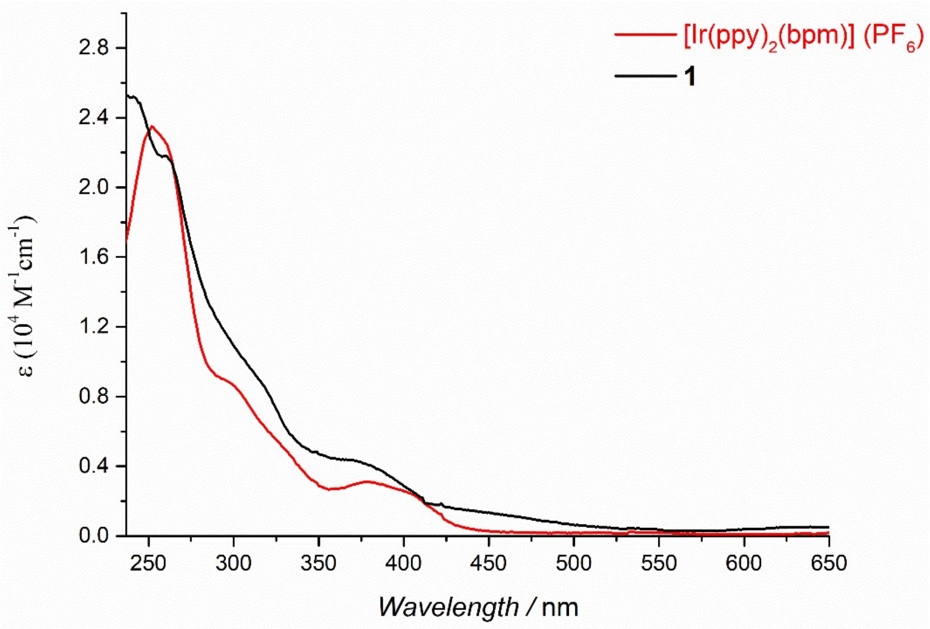

Figure S1. UV-vis spectra of [Ir(ppy)$_2$(bpm)](PF$_6$) and **1** in DCM solution (5×10^{-5}M).

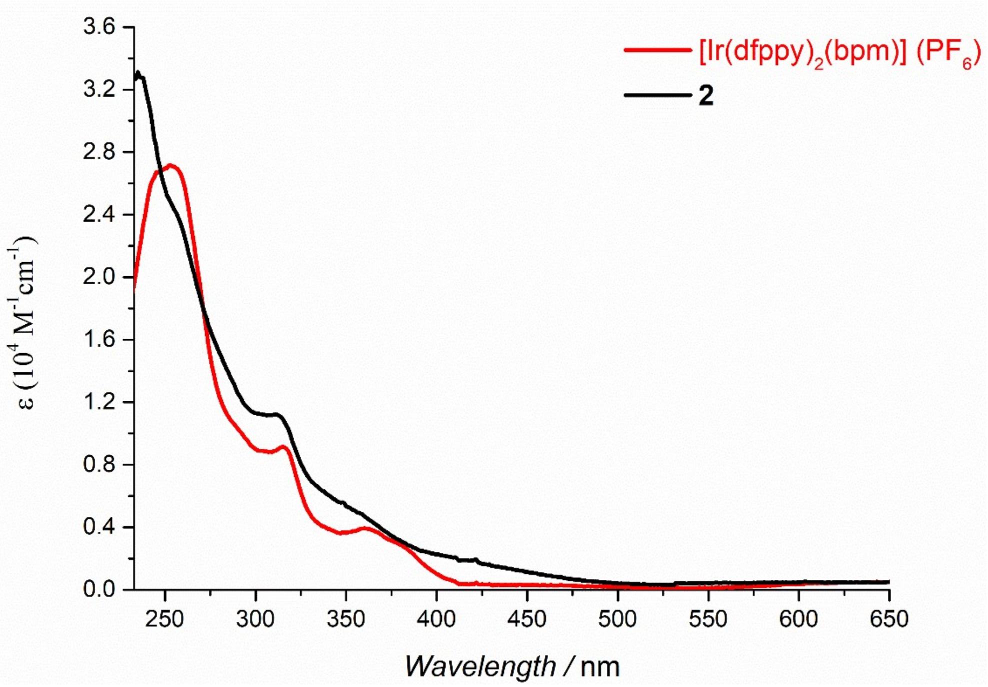

Figure S2. UV-vis spectra of $[Ir(dfppy)_2(bpm)](PF_6)$ and **2** in DCM solution (5×10^{-5}M).

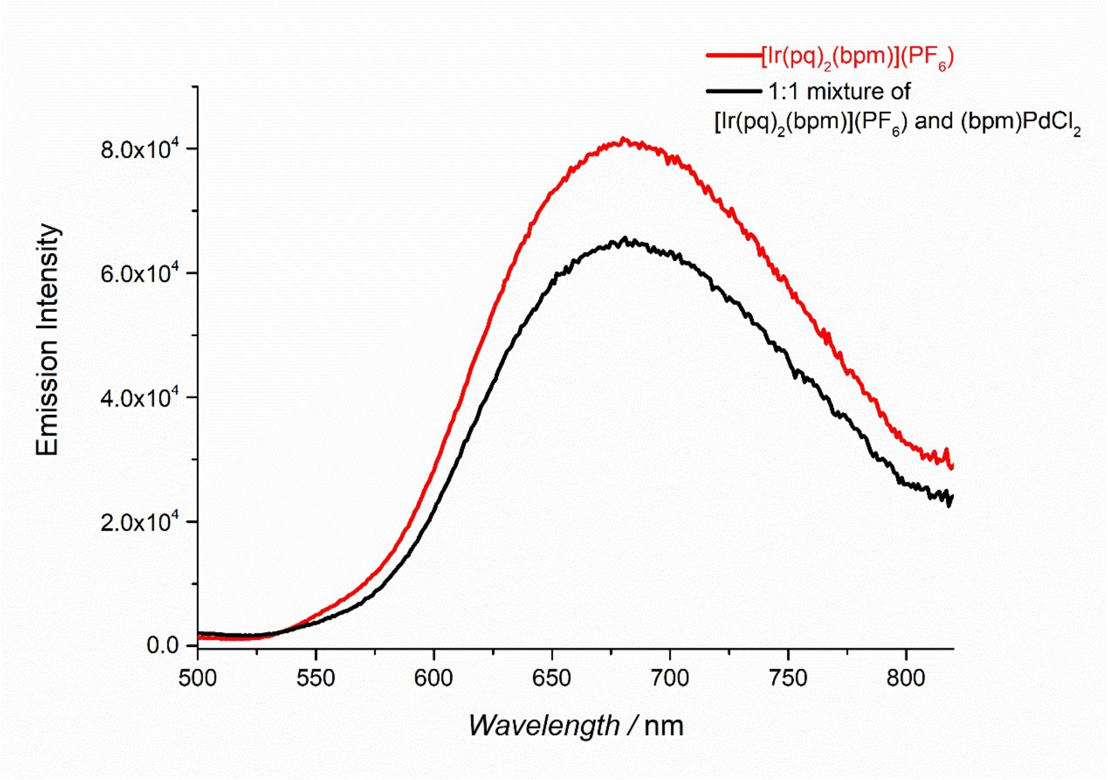

Figure S3. Emission spectra of a mixture of $[Ir(pq)_2(bpm)](PF_6)$ and $(bpm)PdCl_2$ (1:1, 2×10^{-4} M, DCM solution). Excitation wavelength: 405 nm.

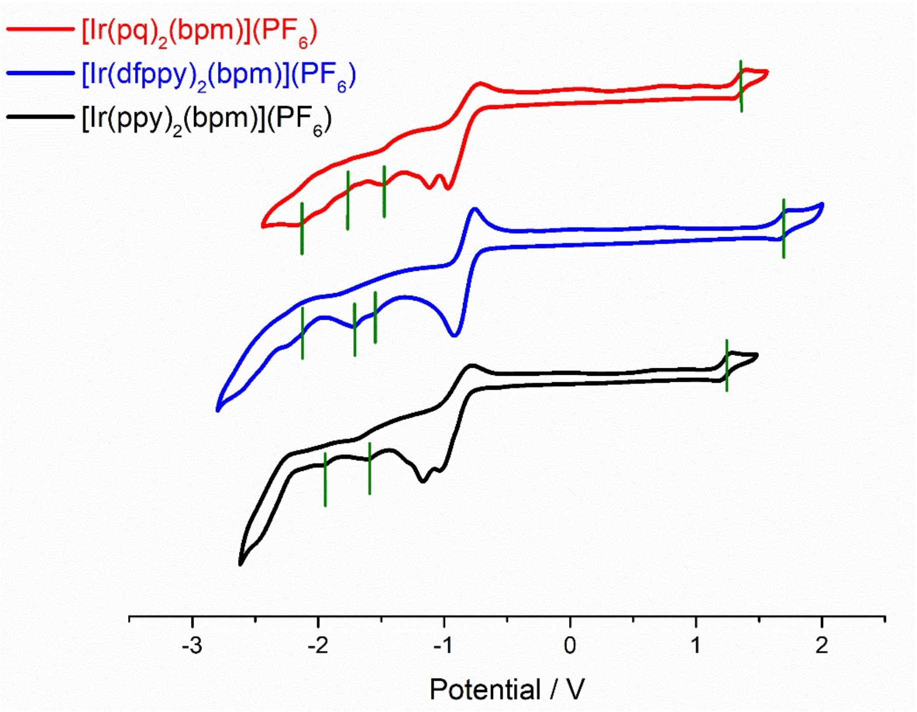

Figure S4. Cyclic voltammograms of mononuclear Ir(Ⅲ) complexes.

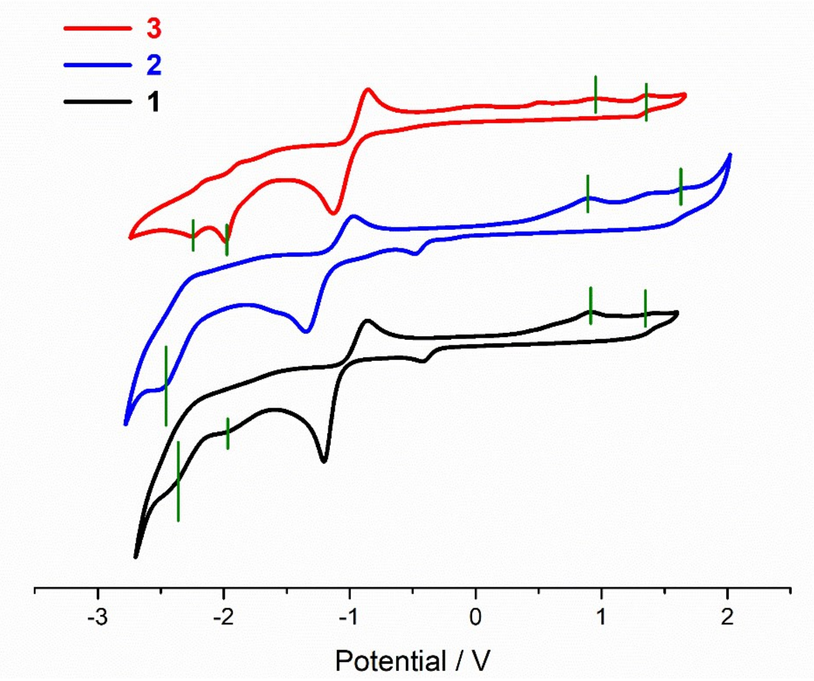

Figure S5. Cyclic voltammograms of Ir-Pd complexes.

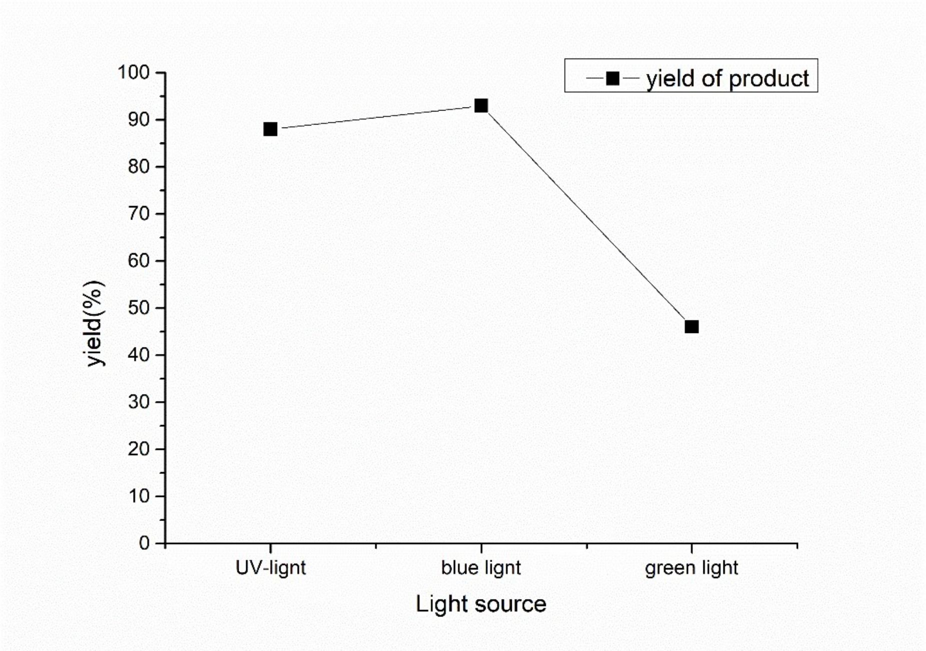

Figure S6. Action spectrum of complex **3**.

Computational Details: DFT calculations were performed using the Gaussian 09W software.[1] Optimization of ground-state structures was performed by using DFT with the B3LYP functional. The LanL2DZ[2a–c] and 6-31G(d,p)[2d,e] basis sets were used to treat iridium and all other atoms, respectively.

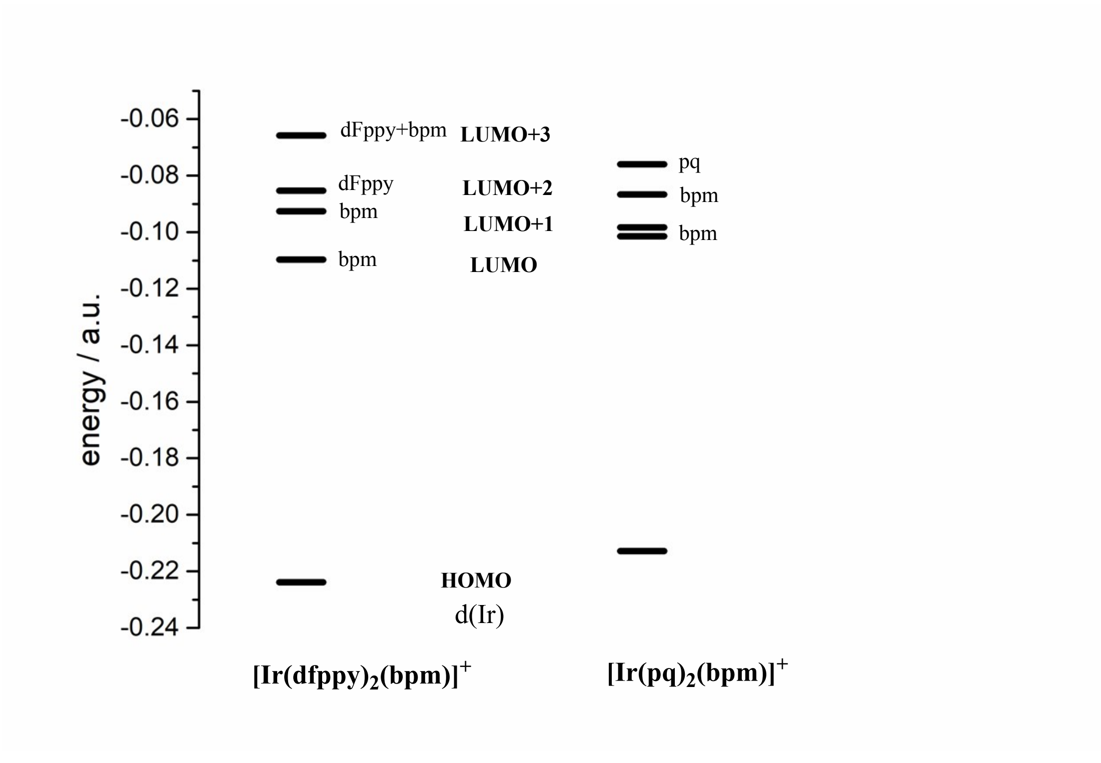

Scheme S1. The energy levels of the frontier orbitals for $[Ir(dfppy)_2(bpm)]^+$ and $[Ir(pq)_2(bpm)]^+$.

^{1}HNMR of catalytical product.

4-Methylbiphenyl: ^{1}H NMR (400 MHz, Chloroform-*d*) δ 7.62 (d, *J* = 7.9 Hz, 2H), 7.54 (d, *J* = 8.1 Hz, 2H), 7.47 (t, 2H), 7.36 (t, *J* = 7.4 Hz, 1H), 7.29 (d, *J* = 7.6 Hz, 2H), 2.44 (s, 3H).

1-(Biphenyl-4-yl)ethanone: ^{1}H NMR (400 MHz, Chloroform-*d*) δ 8.06 (d, *J* = 8.5 Hz, 1H), 7.71 (d, *J* = 8.5 Hz, 2H), 7.66 (d, *J* = 7.1 Hz, 2H), 7.50 (t, 2H), 7.43 (t, 1H), 2.67 (s, 3H).

2-Methylbiphenyl: ^{1}H NMR (400 MHz, Chloroform-*d*) δ 7.45 (t, 2H), 7.40 – 7.35 (m, 3H), 7.29 (dd, *J* = 7.3, 3.3 Hz, 4H), 2.31 (s, 3H).

3-Methylbiphenyl: ^{1}H NMR (400 MHz, Chloroform-*d*) δ 7.63 (d, *J* = 7.1 Hz, 2H), 7.46 (q, *J* = 9.0, 8.1 Hz, 4H), 7.41 – 7.35 (m, 2H), 7.21 (d, *J* = 7.5 Hz, 1H), 2.47 (s, 3H).

4-Fluoro-4'-methylbiphenyl: ^{1}H NMR (400 MHz, Chloroform-*d*) δ 7.56 (dd, *J* = 8.9, 5.4 Hz, 2H), 7.47 (d, *J* = 8.1 Hz, 2H), 7.28 (d, *J* = 7.8 Hz, 2H), 7.14 (t, 2H), 2.43 (s, 3H).

4-(p-Methoxyphenyl)toluene: ^{1}H NMR (400 MHz, Chloroform-*d*) δ 7.55 (d, *J* = 8.8 Hz, 2H), 7.49 (d, *J* = 8.1 Hz, 2H), 7.29 – 7.24 (m, 2H), 7.00 (d, *J* = 8.8 Hz, 2H), 3.88 (s, 3H).

2-Methoxy-1,1'-binaphthyl: ^{1}H NMR (400 MHz, Chloroform-*d*) δ 8.06 – 8.00 (m, 2H), 7.99 (d, *J* = 4.1 Hz, 1H), 7.93 (d, *J* = 8.2 Hz, 1H), 7.68 (t, 1H), 7.50 (d, *J* = 8.5 Hz, 3H), 7.42 – 7.20 (m, 5H), 3.81 (s, 3H).

3,5-Dimethyl-1,1'-biphenyl: ^{1}H NMR (400 MHz, $CDCl_3$) δ 7.63 (d, *J* = 8.3 Hz, 2H), 7.52 – 7.43 (m, 2H), 7.42 – 7.34 (m, 1H), 7.26 (d, *J* = 1.6 Hz, 2H), 7.09 (s, 1H), 2.43 (s, 6H).

2-Methyl-1-phenyl-naphthalene: ^{1}H NMR (400 MHz, $CDCl_3$) δ 7.87 (d, *J* = 7.5 Hz, 1H), 7.81 (d, *J* = 8.4 Hz, 1H), 7.53 (t, *J* = 7.3 Hz, 2H), 7.49 – 7.40 (m, 4H), 7.38 – 7.29 (m, 3H), 2.27 (s, 3H).

2-Methyl-1-(3,5-dimethyl-phenyl)-naphthalene: ^{1}H NMR (400 MHz, $CDCl_3$) δ 7.86 (d, *J* = 8.0 Hz, 1H), 7.80 (d, *J* = 8.4 Hz, 1H), 7.52 – 7.33 (m, 4H), 7.10 (s, 1H), 6.92 (s, 2H), 2.42 (s, 6H), 2.29 (s, 3H).

1-Phenylnaphthalene: ^{1}H NMR (400 MHz, $CDCl_3$) δ 7.94 (d, *J* = 8.2 Hz, 2H), 7.89 (d, *J* = 8.2 Hz, 1H), 7.59 – 7.42 (m, 9H).

2-Methyl-1,1'-binaphthalene: ^{1}H NMR (400 MHz, $CDCl_3$) δ 7.97 (d, *J* = 8.2 Hz, 2H), 7.89 (d, *J* = 8.4 Hz, 2H), 7.66 – 7.60 (m, 1H), 7.54 – 7.46 (m, 2H), 7.40 (t, 2H), 7.33 – 7.14 (m, 4H), 2.13 (s, 3H).

1 M. J. Frisch, G. W. Trucks, H. B. Schlegel, G. E. Scuseria, M. A. Robb, J. R. Cheeseman, G. Scalmani, V. Barone, B. Mennucci, G. A. Petersson, H. Nakatsuji, M. Caricato, X. Li, H. P. Hratchian, A. F .Izmaylov, J. Bloino, G. Zheng, J. L. Sonnenberg, M. Hada,; M. Ehara, K. Toyota, R. Fukuda, J. Hasegawa, M. Ishida, T. Nakajima, Y. Honda, O. Kitao, H. Nakai, T. Vreven, J. A. Jr. Montgomery, J. E. Peralta, F. Ogliaro, M. Bearpark, J. J. Heyd, E. Brothers, K. N. Kudin, V. N. Staroverov, R. Kobayashi, J. Normand, K. Raghavachari, A. Rendell, J. C. Burant, S. S. Iyengar, J. Tomasi, M. Cossi, N. Rega, N. J. Millam, M. Klene, J. E. Knox, J. B. Cross, V. Bakken, C. Adamo,

J. Jaramillo, R. Gomperts, R. E. Stratmann, O. Yazyev, A. J. Austin, R. Cammi, C. Pomelli, J. W. Ochterski, R. L. Martin, K. Morokuma, V. G. Zakrzewski, G. A. Voth, P. Salvador, J. J. Dannenberg, S.Dapprich, A. D. Daniels, Ö. Farkas, J. B. Foresman, J. V. Ortiz, J. Cioslowski and D. J. Fox, Gaussian 09, revision A.1; Gaussian, Inc.: Wallingford, CT, 2009.

2 (a) P. J. Hay and R. W. Wadt, *J. Chem. Phys.* 1985**, 82**, 270-283; (b) R. W. Wadt and P. J. Hay, *J. Chem. Phys.* 1985**, 82**, 284-298; (c) P. J. Hay and R. W. Wadt, *J. Chem. Phys*. 1985, **82**, 299-310; (d) A. D. Becke, *J. Chem. Phys.* 1993**, 98**, 5648-5652; (e) C. Lee, W. Yang and R. G. Parr, *Phys. Rev. B: Condens. Matter.* 1998, **37**, 785-789.
